# Shifting and transforming the practice of audiology: The inclusion of traditional healing

**DOI:** 10.4102/sajcd.v66i1.635

**Published:** 2019-11-20

**Authors:** Dhanashree Pillay, Tshepang Serooe

**Affiliations:** 1 Department of Speech-Language Pathology and Audiology, University of the Witwatersrand, Johannesburg, South Africa

**Keywords:** traditional healers, hearing impairment, integral holistic care model, alternative healthcare, healing

## Abstract

**Background:**

Societal diversity encompasses an array of cultural, religious and spiritual beliefs that influence an individual’s perspective of illness and diseases. Healthcare providers are challenged with the task of considering these diversities in clinical practice. The symbiotic relationship between the healthcare provider and the traditional healer in any healthcare field is rare.

**Objectives:**

The aims were to determine the perspectives of audiologists with regard to traditional healing in South Africa (SA) and to document if and how the audiologist engages with traditional healing in practice.

**Method:**

A questionnaire containing closed and open-ended questions was utilised. Thematic analysis was conducted on the qualitative data, and the quantitative data were displayed using tables and figures.

**Results:**

Forty-one audiologists working at public and private hospitals and clinics in SA were included in this study. The personal experiences of audiologists resulted in varying definitions of a traditional healer. Audiologists reported that patients utilised traditional healing methods such as pouring urine or motor oil into the ear. Strategies of accommodation included being culturally appropriate during conversations, respecting and acknowledging the individual’s cultural and religious beliefs. Twenty-seven audiologists were willing to collaborate with traditional healers to support the patient.

**Conclusion:**

There is a need for an integral holistic model of care in Audiology. There is a lack of communication structures to facilitate the implementation of a collaborative model of care in the current medical model of practice of Audiology. The global trend of holistic and person-centred care is evident, and the field of Audiology cannot negate the role of traditional healers as alternate healthcare providers in SA.

## Introduction

A client describes to the audiologist how a traditional healer played a significant role in the series of events that unfolded after she noticed that her child had a hearing impairment.

The above scenario may vary in its ending. However, the perceptions of the audiologist with regard to traditional healing will ultimately shape the future of the service delivery. So what is traditional healing, and do audiologists engage with traditional healing in South Africa (SA)?

The South African population includes individuals from diverse cultural, religious and linguistic backgrounds. The agenda in SA has shifted from separation and oppression during apartheid to reconciliation and oneness in a democratic society. However, the effects of a deep-rooted apartheid system cannot be instantly overturned. Intentional thoughtful actions are required to ensure that the mandate of transformation occurs within every sector in SA. The existence of diversity does not naturally equate to the acceptance of diversity. Transformation and decolonisation were debated and discussed in length by freedom activists during apartheid as a view towards a democratic SA, also known as ‘Azania’. Post-1994, these discussions became a reality, and South Africans were tasked to implement fair policies and practices to transform the country. Transformation within the healthcare sector was evident as the faces of the healthcare practitioner and the population seeking healthcare were not the status quo of apartheid. Society has a role to play in transforming the present to account for the injustices of the past, and so active participation is warranted in every sector. Thus, understanding the differences in healthcare practices and health-seeking behaviours of the diverse South African population is vital.

The notable diversity of cultures, beliefs and practices between the health practitioner and the individual seeking healthcare calls for attention in the healthcare sector. Traditional healing forms a significant role in the health-seeking behaviours of South Africans, and healthcare cannot negate the impact of traditional healing in the South African context. Traditional healers in SA are synonymous with black African communities; however, the multicultural South African population encompasses racial groups with different traditional, cultural, religious and spiritual backgrounds (Van Vugt & Cloete, 2000).

Audiologists in SA form part of the multidisciplinary healthcare team who provide care that is expected to be culturally and contextually significant to the person receiving care. Traditional healing practices within the current healthcare sector in SA are highly debated because of controversies surrounding the possible adverse impact of traditional healing. Therefore, it was anticipated that this study would seek to determine the audiologists’ views about traditional healing within the dynamic current discourse to transform the service delivery in the healthcare sector in SA.

## Background

Traditional healers have existed for centuries in SA. The forefathers of the different ethnic groups such as African, Indian and Asian groups still rely on traditional healers for spiritual guidance and medical advice (Shapiro & Selin, 2006). The South African Indian population includes Islamic and Hindu healers and religious leaders who perform ceremonies and prayers for the sick using herbs (Ross, 2007). There are three types of South African Muslim traditional healers: the Moulana, who is a spiritual leader who is consulted when there is a psychological, medical and social issue; the Hakeem, who is a Muslim physician; and the gift healer, who is blessed with supernatural powers to provide various treatments for illness and disabilities (Ross, 2007). In the Hindu community, there are different priests and gurus: the Brahman, who is ranked at the top of the castes across India, who occupy the position of priest in the temples of major Gods; and the Sudha Sannyasin and the Yogi, who are respected traditional healers (Ross, 2007). In the Asian community, there are different methods of healing such as acupuncture, herbs and Ayurvedic therapy (Xu & Chen, 2011). Christianity is predominant in SA and individuals who belong to the religion vary in relation to racial and cultural backgrounds. Christians have priests and pastors who are consulted for spiritual support in SA.

Section 1 of the Traditional Health Practitioners (THP) Act (2007a) categorises traditional health practitioners as ‘diviners, herbalists, traditional birth attendants and traditional surgeons’. The Act categorises individuals as per African traditional healing definitions and mandates so that individuals who practise African traditional healing without registering are committing an offence. Since the establishment of the Act, there have been debates pertaining to the inclusion of ethno-medicine, such as hypnotherapy, within the Act. Ms R Ramatsoi, of the National Department of Health, stated at the Traditional Health Practitioners Bill: Public Hearings (2007b) that:

… the categories that were covered in the bill were those that the Committee defined as being based on African medicine traditional practices. Ethno-medicine was not included because it was not entirely based on African traditional medicine. (p. 1)

The Allied Health Practitioners Council of SA accepted ethno-medicine in 2011. However, the 2012 Council reversed the decision as they deemed ethno-medicine to belong under the THP Act (Ethno-Medicine Practitioners Association of South Africa [EPASA], 2015). The application of the Act to traditional healers other than African traditional healers is becoming increasingly moot in SA.

Traditional doctors in SA are also known as *Izinyanga*, who are males who serve an apprenticeship under another *Inyanga* for at least one year and pay their mentors in cash or cows (Meyiwa & Maseti, 2016; Williams, Olopade & Falkson, 2006). Traditional doctors are seen as herbalists, priests and psychologists who can diagnose and treat individuals in a direct manner as would a medical doctor (Davids et al., 2014). Diviners are also known as *Izangoma*; they are usually females and only people who have a calling from the ancestors can become one (Ogana & Ojong, 2015). An *Isangoma* can have a consultation with the family without the individual being there, and through the use of spiritual insights, these Diviners can interpret the cause and consequence of the illness, and they are capable of determining the cause of the problem or illness by consulting ancestral spirits (Ross, 2010). The Faith Healers who are also known as *Abathandazi* are professed Christians, who belong to one of the independent African churches, and they heal by prayer, holy water or ash, and the laying on of hands on the individual who needs healing (Ross, 2010). Race, spirituality and religion are not closed groups of individuals, and healthcare professionals in SA are guaranteed to encounter the diversity and intertwining of beliefs and practices.

Individuals in SA consult different types of traditional healers for various reasons such as protection against witchcraft, illnesses, their ability to prophecy about future events, divulgence of secrets and annual check-ups (Ross, 2010). Research pertaining to traditional healing has predominantly focused on conditions such as mental health (Ae-ngibise, Coopet, Adiibokah, Lund, & Doku, 2010), Acquired Immune Deficiency Syndrome (AIDS) (Somse et al., 1998), tuberculosis (TB) (Colvin, Gumede, Grimwade, Maher, & Wilkinson, 2003) and sexually transmitted infections (STIs) (Peltzer, Mngqundaniso, & Petros, 2006). Studies in relation to communication impairments and traditional healers have focused on the areas of cleft lip and palate, stuttering and aphasia (Dagher & Ross, 2004; Platsky & Girson, 1993; Legg & Penn, 2012). Research conducted in the field of audiology focused on the perspective of traditional healers regarding hearing impairments (De Andrade & Ross, 2005) and the training that traditional healers obtain in relation to hearing impairments (De Andrade, 2011). The South African population consults traditional healers for different illnesses including hearing loss (De Andrade, 2011; Summerton, 2006), and the audiologist cannot negate the impact of traditional healing within the biopsychosocial sphere of the individual.

People consult traditional healers for hearing impairments, and the traditional healers have various ways of managing the individual (De Andrade & Ross, 2005). In a South African study, the management of a hearing impairment by traditional healers included animal- and plant-based remedies, and rituals such as the laying on of hands to treat the hearing problems (De Andrade & Ross, 2005). One of the major concerns surrounding treatment methods of the traditional healer is the dispensing of remedies into the ear even when the tympanic membrane is ruptured, which may result in further middle ear complications (Snow, Wackym & Ballenger, 2009). Some traditional remedies such as skanama and matekwane (marijuana) may also result in memory problems, damage to internal organs such as the lungs and cerebral atrophy (Kaplan & Sadock, 1991). The collaboration between the audiologist and traditional healers may prevent damage and ensure that the individual is supported within an integrated and holistic model of service delivery in healthcare. Audiologists can gain insight and knowledge into the principles and foundations of the traditional healers’ treatment methods that will enhance the healthcare service delivery system.

Traditional healers in SA indicate that they are willing to collaborate with the medical professionals (Champbell-Hall et al., 2010; De Andrade & Ross, 2005). However, studies revealed that the medical professionals were not willing to collaborate with the traditional healers (Mills et al., 2006; Peltzer et al., 2006; Somse et al., 1998). Medical professionals are unwilling to collaborate with the traditional healers because of a lack of scientific proof of the traditional practices (De Andrade & Ross, 2005). In the 1970s, the WHO called for the inclusion of traditional health practitioners in the provision of primary healthcare and for the social and technical training of traditional health practitioners in an effort to include traditional health practitioners in health promotion activities (WHO, 1978). The South African Traditional Health Practitioners Act (2007a) attempted to bring principles of regulation into traditional practice. However, the Doctors for Life group, which represents over a thousand medical professionals in SA, objected to the government’s plan to legitimise the traditional healers (Madomombe, 2006). The group objected to the plan as traditional healers are not validated scientifically, and many individuals experience complications because of the use of traditional medicines (Madomombe, 2006). The Doctors for Life group also believed that this law would result in legal controversies and medical complications. The group urged that all the traditional remedies should be thoroughly researched before being approved. Even though the Doctors for Life group objected to the government’s plans to legitimise the traditional healers, there were some medical professionals that pointed out that with or without the support of the law, traditional healers are already providing services in many communities. Therefore, these medical professionals supported the inclusion of traditional healers within primary healthcare for a collaborative environment between the two service providers (Madomombe, 2006).

Despite the unwillingness of the medical professionals to collaborate with traditional healers, many South Africans continue to consult both the traditional healer and the medical professional for various reasons (Champbell-Hall et al., 2010). There is a need to understand the spiritual, cultural and religious beliefs of the individual with a hearing loss within any context. Audiology is a branch of science and medicine which focuses on the sense of hearing and balance (Johnson & Seaton, 2011). The humanistic aspect of healthcare must include the social element in the service delivery model. Audiology in SA traditionally operates under the principals of the medical model, which focuses on clinical medicine that is based on the principles of the natural science. The audiologist utilises western methods of care that focuses on the individuals’ biological processes rather than their social, emotional and spiritual processes (Alderfer, 2010). In contrast, the traditional healer uses various substances and methods based on social, cultural and religious belief systems that are not scientifically proven (Albertyn, Berg, Numanoglu & Rode, 2015), yet the South African population still chooses to consult traditional healers. The areas of science and spirituality have been seen as separate entities in healthcare, and specifically in audiology, for far too long.

There is a desire and willingness from the traditional healers in SA to collaborate with the audiologist (De Andrade & Ross, 2005). However, there is a dearth of information pertaining to the audiologists’ perspective. It is essential to bridge the gap between the traditional healers and audiologists to ensure that the services provided to the individual with a hearing loss are culturally and contextually appropriate to meet the needs of the dynamic and diverse South African society. This study assists in filling the gap that pertains to the audiologists’ perspective in relation to the possibility of an integral and holistic model for collaborating with the traditional healers in SA.

Will audiologists continue to be shaped by the western medical model of care, or are audiologists entering a new era of change towards a more holistic integral model of healthcare?

## Research method and design

### Aims of the study

- To determine the perspectives of audiologists with regard to traditional healing in SA.
- To document if and how the audiologist engages with traditional healing in practice.

### Research design and instrumentation

A non-experimental cross-sectional survey research design was utilised in this study. A self-developed questionnaire utilising open- and closed-ended questions provided qualitative and quantitative data. The researchers developed a questionnaire that was electronically distributed to all participants.

### Sample and sampling

Purposive sampling was used to recruit participants from the online healthcare association registers in SA. Participants were required to be registered with the Health Professions Council of South Africa (HPCSA) and had to be practising in SA. This study also included audiologists who are dually trained as speech therapists who work within the public and private healthcare sectors in Gauteng. Male and female participants of different ages were included in this study. Questionnaires were electronically sent to 100 participants; 52 participants agreed to participate in this study. However, only 41 participants completed the questionnaire. The detailed responses in this study are worth noting despite the 41% response rate. The quality of responses should not be ignored. However, a larger sample size would provide greater generalisation of findings.

### Ethics and data analysis

Thematic content analysis was used to analyse the open-ended questions. The closed-ended questions were analysed using quantitative methods. Data were grouped and displayed descriptively using tables and figures.

### Ethical considerations

Ethical clearance was granted by the University of the Witwatersrand’s, Non-Medical Ethics Committee on 16 April 2016 (clearance number: STA_2016_01). Informed consent was obtained from each participant prior to answering the questionnaire. Anonymity and confidentiality were adhered to for all participants.

## Results

### Participant demographic information

Forty-one participants comprising of 38 female, two male and one other, who chose not to identify with either male or female, participated in this study. The majority of participants were Christian (20).

English was the first language of the majority (24) of participants as per Table 1. The participants had different years of experience as a practising audiologist as seen from Table 1. Participants had completed their Audiology degree at either the Medical University of South African (MEDUNSA) (2), University of the Witwatersrand (Wits) (19), University of Pretoria (UP) (7), University of Cape Town (UCT) (3), University of KwaZulu-Natal (UKZN) (7) and the University of Stellenbosch (1), and four participants did not state where they had completed their degree in Audiology.

**TABLE 1:** Participants’ demographic information.

Demographic factors	Subcategory	Number
Gender	Female	38
	Male	2
	N/A	1
Religious affiliation	Christian	20
	Hinduism	5
	Islamic	3
	Judaism	7
	Traditional African Religion	0
	Atheist	1
	N/A	6
First language or Home language	Afrikaans	5
	English	24
	IsiNdebele	0
	Sepedi	1
	SiSwati	1
	Sesotho	1
	Xitsonga	0
	Setswana	0
	Tshivenda	0
	IsiXhosa	0
	IsiZulu	3
	Kiswahili	1
	Creole	1
	N/A	4
Number of years practising as an audiologist	1–5	13
	6–10	16
	11–20	8
	21–35	3
	N/A	2
University where participant completed the Audiology degree	MEDUNSA	2
	Wits	19
	UP	7
	UCT	3
	UKZN	7
	SU	1
	N/A	4

MEDUNSA, Medical University of South African; UP, University of Pretoria; UCT, University of Cape Town; UKZN, University of KwaZulu-Natal; SU, University of Stellenbosch; N/A, Not Applicable.

### South African audiologists’ description of a traditional healer

A traditional healer was described as:

‘Traditional does not equate to bad, backward, worse, negative – it’s just ‘different’ to the imposed ‘normal.’ (P1, female, audiologist)

‘A traditional healer includes individuals who have not studied a medical degree. They generally use alternative medicines. They are frequently portrayed as using voodoo including spells.’ (P2, no gender, audiologist)

‘A person that uses their cultural and or religious beliefs for healing the ill.’ (P8, female, audiologist)

‘Someone who sees themselves as being called to help others, in terms of healing from physical, emotional or psychological illnesses or diseases. They do not have any official and/or academic qualifications, it is more a calling.’ (P9, female, audiologist)

‘Someone who functions in a community … makes use of healing methods that are not necessarily supported by medical research … often thought to be selected by a spiritual presence.’ (P10, female, audiologist)

‘In the South African setting I view a traditional healer as the colloquially called Witch Doctor.’ (P12, female, audiologist)

‘Provides medical attention to their community through traditional methods that have been passed down through generations … the community respects their knowledge about illnesses and problems … can have a coexisting training in modern medicine but do not solely rely on that training when treating patients.’ (P29, female, audiologist)

Table 2 presents the examples of traditional healers that were provided by participants.

**TABLE 2:** Examples of traditional healers.

Example of traditional healers provided by the participants	Number of participants
Sangoma	27
Homeopath	3
People practising Chinese medicine	2
Reiki master	1
Acupuncturist	1
Rabbi priest	1
Religious leaders	1
Witch doctor	3
Herbalist	5
Iridologist	1
Spiritual people or elders	1
Individuals who treat medical problem without a medical degree	1
Inyanga/Nyanga	6
Ayurvedic healers	1
Moulana	1
Individuals with trance	1
Respected individual in the community with appropriate skills/trained by previous traditional healer	2
Ministers	1
Umthandazi	2
Indian medicine people	1
Traditional healers who are in the medical professional, dually trained and practising (dentist, nurse)	2
Traditional healer who also works in government	1

Two participants stated that traditional healers may have coexisting training in modern medicine, but they do not solely rely on that training when treating clients. Three participants stated that consultations with traditional healers are common in the specific racial groups in SA. Participants stated that traditional healers could also be spiritual healers from other cultural and religious groups, such as the Muslim, Jewish, Hindu and Chinese groups.

### South African audiologists’ engagement with traditional healing

#### Do audiologists ask about traditional healing?

Thirteen participants reported that they ask about traditional healing during the case history or the feedback session. Two themes were identified, which show that asking about traditional healing:

- facilitates an understanding of the clients’ belief system
- provides insight into the health-seeking behaviour of the clients, prior to seeing the audiologist.

Audiologists ask about traditional healing to gain knowledge and understanding of the client in a holistic manner. The quotes pertaining to the above themes include:

‘It gives me an idea of what the patient’s belief system is, what their health seeking behaviours are, and how they will respond to my intervention.’ (P1, female, audiologist)

‘I ask what have you done or who have you been to see, to help with the problem. This is generally when patients will offer information and then I will probe further.’ (P11, female, audiologist)

‘I have asked about it when I worked in state hospitals and for research purposes. The use of traditional healers can sometimes conflict with our practice, and it is important to ascertain what the patient believes in.’ (P13, female, audiologist)

‘Often African people going to the traditional healer first, is not taboo. And often patients come to hospitals only when the traditional route has failed. As an audiologist this often came up at the end of the session but now it’s part of the first few questions I ask about especially for patients with discharging ears or unknown sudden hearing losses.’ (P2, non-gender, audiologist)

‘For counselling purposes. Most patients don’t believe in congenital hearing loss. So they go to traditional healers first before they seek any rehabilitation or medical attention.’ (P34, female, audiologist)

Twenty-eight participants reported that they do not ask about traditional healing during the case history and feedback sessions, and one participant reported that he or she asks occasionally. Themes that were identified were:

- Information is not deemed to be relevant.
- Information is deemed to be intrusive.

Some participants have never considered asking about traditional healing as it is not within their scope of practice, and they have asked only direct medical questions pertaining to the ears. The reasons for not asking about traditional healing include:

‘It generally would not affect the patient’s hearing. I would only ask if the patient mentioned having been to a traditional healer or had a traditional healer suggest to them that their hearing loss may be caused by angry spirits/ancestors.’ (P5, female, audiologist)

‘I feel like this information needs to be volunteered by the client should they wish to share this information.’ (P6, female, audiologist)

‘It feels too intrusive into someone’s traditions and religious beliefs.’ (P9, female, audiologist)

‘It doesn’t seem relevant in my population of patients/context.’ (P15, female, audiologist)

‘Never thought about it.’ (P24, female, audiologist)

‘I don’t ask about traditional healing directly, I ask more specifically if they have received any form of assessment or treatment from anyone regarding their ears. Such as for pain, noises in the ear or anything else related to the ears and hearing.’ (P29, female, audiologist)

‘It’s a sensitive topic.’ (P31, female, audiologist)

I have never come across a situation where I have to, but patients have enquired about them on treatment options.’ (P36, female, audiologist)

#### Do audiologists receive client reports of traditional healing?

Nineteen participants reported that clients have never informed them about consulting with a traditional healer. Participants, who received a report, stated the following accounts:

‘[*The parents*] took the child to an Inyanga after the child lost his hearing post meningitis. The Inyanga recommended she come to the hospital.’ (P2, no gender, audiologist)

‘Unfortunately, the traditional healer let the client wear a cord around his stomach. The traditional healer also threw red and blue beads into the client’s ears. It did not work for the client’s condition.’ (P9, female, audiologist)

‘The tongue was stuck to a button and it was released through surgery but she [*the mother*] was disappointed as the child didn’t learn to talk thereafter.’ (P11, female, audiologist)

‘A young boy with meningitis having his hair shaved in the form of a cross to heal him. It did not help him. He nearly died.’ (P12, female, audiologist)

‘A patient told me he was advised to put motor oil into his child’s ears.’ (P17, female, audiologist)

‘Making small lacerations on the chest for tuberculosis [TB] treatment. Pouring oil and herbs in the ear to assist with infections or hearing loss.’ (P18, female, audiologist)

‘The patient was diagnosed with spinal tuberculosis and meningitis and the patient’s family was burning herbs in the ward. The patient was provided with traditional healing medication and was not taking the prescribed medication from the doctor.’ (P23, female, audiologist)

‘They mentioned that they did go see an Isangoma, as the patient believed that someone from the community performed black magic causing her to lose her hearing. Recommendations suggested that she drink something that was made up by the Isangoma.’ (P28, female, audiologist)

‘The healer instructed her to put her own son’s urine into his ear to help with the pain and infection.’ (P29, female, audiologist)

‘They saw a prophet and had to pay a lot of money to them, their child still remained profoundly deaf afterwards. (P37, female, audiologist)

‘This has happened many, many times – more than 50 times. The traditional healer was almost always consulted to provide input or treatment for an adult or child who had lost their hearing or was ill … the client was told that the afflictions were a message from their ancestors, and this did give them some peace, having what they believed to be a cause for the difficulty. In several cases, the treatment actually caused further medical concerns.’ (P40, female, audiologist)

#### Are audiologists accommodating of the client’s cultural and religious beliefs in practice?

Two participants stated that they do not accommodate their clients’ cultural and religious beliefs, and 39 participants are accommodating as per Box 1. Participants stated that they accommodate their clients by not forcing intervention (e.g., the use of hearing aids). They ensure that communication style is culturally appropriate, and they try to communicate with the client in the client’s first language.

BOX 1 Strategies used by audiologists to accommodate the client’s cultural and religious beliefs.

**Things that the audiologist does to accommodate the client’s cultural and religious beliefs**

- Checking with the client to determine if procedures are culturally appropriate.
- Respecting and acknowledging the client’s cultural and religious beliefs, and not imposing his or her own beliefs on the client.
- Informing the client that they are entitled to a second opinion.
- Greeting the client and trying to conduct case history or feedback in his or her first language, or at least using layman terms, but avoiding talking down to the client. Getting an interpreter.
- Not forcing a client to participate in any form of treatment he or she is uncomfortable with.
- Does not specifically ask about consulting a traditional healer or using alternative methods, but listens to and respects the client’s views if he or she shares this information.
- When a client states that he or she wants to see the traditional healer first, the audiologist should counsel the client with regard to time sensitivity (especially when the client is a child). Ask for consent before conducting any procedure.

Participant 22 was the only audiologist to describe a direct interaction with a traditional healer while consulting with a hearing impaired individual:

‘I had a patient actually come to see me with his traditional healer. He had a long history of discharging ears and poor functional hearing. He had gone to this lady for assistance. She (the traditional healer) at some point realised that her medication would not work and recommended that he should see a doctor at a clinic. It was quite interesting as I explained what otitis media is and how it manifests and how it should be treated with antibiotics and medical treatment, and how the ear needs to be kept dry as one of her treatment plans was inserting water with stuff in. They were quite receptive and the traditional healer said she would pass the message on to her colleagues, which was encouraging to hear.’ (P22, female, audiologist)

#### Collaboration between traditional healers and audiologists

Thirty-nine participants reported that they have never received a referral from traditional healers, one participant reported that he or she had received a referral from a traditional healer and one participant did not answer this question.

Twelve participants did not answer the question pertaining to a referral to a traditional healer, one participant stated he or she has never been in a situation to refer, 27 participants reported that they would not refer the client to a traditional healer, and one participant stated that he or she would refer. Themes that were identified included:

- the lack of scientific evidence to support traditional healing
- personal beliefs of the participant.

Participants stated that they would not refer the client to traditional healers because the methods are not scientifically proven. Participants indicated that traditional healers are not qualified medical professionals, and if something goes wrong, the audiologist who has made the referral may be held responsible. Four participants stated that traditional healing is not within the medical scope of practice; therefore, they would rather refer to other medical professionals. Participants also stated that this would be against their own beliefs, as they do not believe in traditional healing. However, there were some participants who stated that if the client has psychological or emotional issues that could be addressed by the clients’ spiritual leaders, they would ‘let’ them consult with a traditional healer. When asked about a referral to a traditional healer, the following answers were provided:

‘No. I’m not versed in traditional healing; I don’t have evidence of its efficacy, so I would not refer a patient to them for audiological management.’ (P1, female, audiologist)

‘My faith may interfere especially if they say one was bewitched.’ (P3, female, audiologist)

‘No I don’t know any traditional healers.’ (P4, female, audiologist)

‘No I don’t believe in alternative medicine.’ (P21, female, audiologist)

‘No not part of my belief system.’ (P25, female, audiologist)

‘No. Never. If there is no audiological or medical basis for the patients complaint, the patient is counselled and discharged. In some cases I have referred the patient to a psychologist.’ (P33, female, audiologist)

One participant stated that she would refer clients to traditional healers:

‘Not yet but if I encountered such a case I would. Reason being I, myself believe in alternate treatments as well as the evil eye.’ (P16, female, audiologist)

Twenty-seven participants stated that it would be beneficial to have a formal model for the collaboration with traditional healers as the majority of the South African population consults with traditional healers. The themes that were identified include:

- Collaboration supports holistic client care.
- There are clear boundaries for collaboration.

A participant stated that the collaboration should begin with educating each group about the other, as this would lead to a multidisciplinary team that accommodates the clients’ beliefs. There were participants who were against the idea of collaboration as they deemed it ‘confusion’. They stated:

‘As long as the traditional healer is a Christian and uses herbs and no witchcraft.’ (P3, female, audiologist)

‘There is room for both views and understanding both views are important for the sake of the client.’ (P6, female, audiologist)

‘I believe there is a big gap in collaboration. And perhaps that lies within our ignorance as healthcare professionals. Perhaps we do not know enough about traditional healing to make the appropriate referral. Perhaps our views have been influenced by the stereotype of what we think traditional healing is. An idea would be to start with education – from both sides … Perhaps if traditional healers include medical research into their practice, their practice would become safer/more medically recognised.’ (P10, female, audiologist)

‘Traditional healers are central to how certain communities run. They carry a lot of power. They are often the first place parents go for help when they notice a problem. This collaboration could assist in early identification and intervention.’ (P11, female, audiologist)

‘I think there is great power in collaborating and sharing ideas.’ (P12, female, audiologist)

‘I don’t know the role of traditional healers and would be nervous about this.’ (P15, female, audiologist)

‘I don’t think it’s something I would do.’ (P18, female, audiologist)

‘It is important to consider the patient holistically and take their needs into consideration. Traditional healers might be able to tap into areas that medical practice is not able to tap into.’ (P23, female, audiologist)

## Discussion

In this study, the general consensus is that a traditional healer is well respected in the community with a calling to help others through non-medical means. A vital finding in this study is that traditional healers are thought to use herbal remedies, surgery, prayer, spiritual rituals and practices to heal a physical, emotional or psychological illness or disease. The participants stated that the traditional healer does not have an official or academic medical qualification but they treat medical illnesses and diseases. South African audiologists perceived the traditional healer’s methods to be unscientific, which reiterates the medical western view of traditional healing practices. Participants perceived that traditional practices may sometimes have benefits to improve the client’s health, but they may become dangerous or contradictory when used with modern or western medicine. The methods are dictated by the cultural and religious beliefs, which have been passed on from generation to generation through ancestors. The descriptions of a traditional healer that were provided by participants were similar to the WHO classification of a traditional healer. Traditional healers are well-respected people in their respective communities. However, the participants did not see traditional healers as equals or as medical professionals because they do not have the academic qualifications and their methods are not scientifically proven. Traditional healing is based on spirituality which may result in conflict and misunderstandings between the traditional healer (Colvin et al., 2003) and the western medical professional, whose point of view is based on scientific proof (Poudyal et al., 2003).

The participants in this study stated that they do not ask about traditional healing because it is a very sensitive topic that seems intrusive, highlighting that audiologists are reluctant and possibly unequipped to appropriately converse about aspects such as spirituality and traditional healers within the healthcare practices. Based on the HPCSA 2008 guidelines for good practice in healthcare, it is the healthcare worker’s duty to have the client’s well-being and best interest at focus. The healthcare worker has a duty to make sure that the client’s personal beliefs do not cause any harm or injury (HPCSA, 2008). Therefore, an integral and holistic model of care is vital to ensure that all facets of the client’s life are included in the care process.

The results of this study revealed that audiologists have encountered negative aspects of traditional healing practices that compromise the client’s health as some clients were reluctant to take the medical treatment when they were taking the traditional medication, and the methods that were reported by the participants seem unconventional when compared to western medical management. In SA, some traditional healing methods may cause harm to the client (De Andrade & Ross, 2005). However, there are other international studies (Ross, 2010) that reveal the benefits of traditional healing methods. This study indicates the need for prevention and promotion of the scope of practice of western medical professionals and traditional healers and the implementation of an integral model of care, without contradictions. It is evident that audiologists must consider the inclusion of questions that pertain to spirituality and traditional practices to ensure that models of care are culturally and contextually appropriate for the South African population.

When the healthcare provider initially establishes rapport with his or her client, the client develops a trust relationship with the healthcare provider. The clinician who builds a trust relationship with the client can motivate for compliance when implementing intervention (Hegde & Davis, 2009). The South African society is multidimensional, and therefore, an integrated holistic service delivery model is vital in the practice of audiology. A second opinion is a free choice, and it is a right afforded to the client; however, the medical model has shaped this right to suit only a medical second option. It is evident that the majority of audiologists do not refer to traditional healers, and they do not receive referrals either. Traditional healers in other studies were willing to collaborate with the western doctors; however, they acknowledged that the medical healthcare providers do not respect traditional healing methods (Madomombe, 2006). The transformation of clinical and academic training of audiologists in SA must include information that focuses on cultural and religious beliefs within the curriculum.

The advantages of collaboration can be a holistic and integral service delivery model that cares for an individual with a hearing loss and his or her family. The biopsychosocial-spiritual model assists in considering the client’s biological factors, psychological factors, social factors and spiritual factors (Sulmasy, 2002). Internationally, and within SA, there are still debates about the inclusion of traditional healers in the healthcare system because of their non-scientific methods of healing (Colvin et al., 2003). However, there is evidence of successful collaborations between the medical and traditional worlds (Chipfakacha, 1997; Peltzer et al., 2006; Pillay & Moonsamy, 2017, 2018; Poudyal et al., 2003; Somse et al., 1998). Tools that may aid in promoting integral healthcare, such as the Faith and Beliefs, Important, Community and Assess in Care (FICA) and Source of Hope, Organized Religion, Personal Spirituality and Practice, Effects on Medical Care (HOPE), could assist the healthcare provider in introducing spirituality into the session (Saguil & Phelps, 2012).

## Conclusion

The foundations of service delivery and practice in audiology have been solely based on the medical model of healthcare. The illness- and disease-centred model is effective as it assesses and manages the hearing loss; however, it negates the humanistic aspects related to the individual’s experiences. This article opens a door to discussing the audiologists’ perspectives of traditional healing, which plays a significant role in the field of audiology in SA. The pertinent findings in this article highlight the real-life experiences of South African audiologists. Collaboration with traditional healers may unlock the possibility for formalising an integrative holistic model of healthcare service delivery in audiology. Audiologists are starting to accommodate clients’ beliefs during consultations. However, there is uneasiness when considering collaborations with traditional healers. The lack of knowledge about the scope of practice of both the audiologist and the traditional healer must be dealt with to ensure that holistic and integrated care is provided to the individual with a hearing loss.

With reference to holistic healthcare, it is recommended that audiologists consider the following:

- The audiologist should reflect on personal beliefs, thoughts and assumptions that impact one’s practice. A self-reflection session may include questions such as:
  ■ How is ultimate health understood?
  ■ How do you understand affliction and suffering?
  ■ What are the different parts of a person?
  ■ How is hearing loss understood and explained?
  ■ What intervention and care are seen as necessary?
  ■ What do you understand by the word ‘healing’?
  ■ Who is seen as qualified to address the different parts that need healing?
  ■ What are your thoughts on religious, traditional and spiritual care in general and hearing loss in particular?
  ■ Do you believe in traditional healing, and if you do not believe, then how would you handle a situation where a patient believes in traditional healing?
  ■ What are your thoughts with regard to the collaboration with non-medical individuals in the management of a hearing loss?
  ■ Would you consider initiating a multidisciplinary team that includes religious, spiritual and traditional healers in the care of an individual with a hearing loss?
- The audiologist is advised to re-evaluate the history-taking process and consider the inclusion of questions pertaining to religious practices and belief systems. The audiologist may consider asking the following questions:
  ■ Do you believe that the hearing loss may be a result of a non-medical cause?
  ■ Do you believe in God or a Higher Power, and if so, how do you understand the hearing loss in relation to your belief?
  ■ Have you sought any other form of help for the hearing loss, for example, prayer, traditional methods?
  ■ Is there someone who supports you spiritually?
  ■ Would you like to include non-medical support structures in the process of management of the hearing loss?

The authors leave you with the challenge of exploring your own spiritual, religious and traditional beliefs so that you may afford the client with the same opportunities.
